# Frequency and risk factors of carotid atherosclerosis in patients with coronary artery disease

**DOI:** 10.1186/s12872-025-05501-1

**Published:** 2026-01-27

**Authors:** Mohamed El-feky, Sherif Aboushara, Amr Zaki, Mohamed Loutfi, Ahmed Elhfnawy

**Affiliations:** 1 Department of Cardiology, University of Alexandria, Alexandria, Egypt; 2 Department of Neurology, University of Alexandria, Champollion street, Al Mesallah Sharq, Al Attarin, Alexandria Governorate, Alexandria, 5372066 Egypt

**Keywords:** Coronary artery disease, Atherosclerosis, Carotid stenosis, Syntax score

## Abstract

**Background:**

Carotid Atherosclerosis is more prevalent among patients with coronary artery disease (CAD). To the best of our knowledge, research on the prevalence of carotid atherosclerosis in CAD patients is mainly based on retrospective cohorts or registries. We aimed to investigate the frequency of carotid atherosclerosis among patients with angiographically proven CAD and to investigate, whether carotid atherosclerosis is more prevalent in special subgroups.

**Methods:**

We recruited consecutive patients with angiographically proven CAD, who presented to our cardiology department with manifestations of ischemic heart disease. Carotid atherosclerosis was assessed using ultrasound.

**Results:**

We included 103 patients in our study. Eighty-three (80.6%) patients were males, the mean (± SD) age was 58 (± 9) years. Carotid atherosclerosis was detected in 74 (71.8%) patients. Of those, 6 patients (5.8%) had ≥ 50% stenosis. The ROC curve showed that a syntax score > 17 had a sensitivity of 64.9% and a specificity of 72.4% for carotid atherosclerosis (AUC 0.68, 95% CI 0.57–0.79, *p* = 0.006). Using a multivariate binary logistic regression after adjusting for age and sex, we found that age ≥ 60 years (OR 5.1, 95% CI 1.64–15.86, *p* = 0.005), male sex (OR 3.61, 95% CI 1.03–12.67, *p* = 0.045) and ≥ 3-vessel CAD (OR 4.18, 95% CI 1.06–16.51, *p* = 0.04) were associated with carotid atherosclerosis. In another age- and sex dependent multivariate logistic regression, syntax score > 17 independently predicted carotid atherosclerosis (OR 4.51, 95% CI 1.68–12.16, *p* = 0.003).

**Conclusions:**

It seems to be reasonable to conduct carotid ultrasound screening for patients with CAD, especially older males with high syntax score and/or ≥ 3-vessel CAD.

## Background

Atherosclerosis is the most common cause of death worldwide [1]. Atherosclerosis is a systemic disease that affects multiple vascular beds including the coronary vessels causing ischemic heart disease as well as the carotid arteries posing the patients at risk of ischemic strokes [1, 2]. Myocardial infarction, stroke, vascular mortality, or rehospitalization rates at three-year follow-up are 40.5% for patients affected by symptomatic disease in multiple arterial systems and 25.5% for patients with symptomatic disease in one arterial system [3]. Furthermore, high burden of carotid plaques is an important predictor of new-onset myocardial infarction [4]. Over the past decades, vascular mortality has significantly decreased in high-income countries [5]. However, vascular mortality did not dramatically decline in low- and middle-income countries [5]. Routine screening for asymptomatic carotid atherosclerosis is not recommended in the general population [6]. Nevertheless, among patients with multiple risk factors, screening for carotid atherosclerotic disease may be considered [7]. Ultrasound is a reliable cost effective method to assess carotid atherosclerosis [8]. Increased carotid intima-media thickness as well as carotid plaque burden are more prevalent among patients with coronary atherosclerotic disease (CAD) [9, 10]. To the best of our knowledge, studies regarding the prevalence of carotid atherosclerosis among patients with CAD are mostly based on registries or retrospective cohorts. In the current work, we aimed to investigate the frequency of carotid atherosclerosis in a sample of patients with angiographically proven CAD and to find out, whether carotid atherosclerosis is more prevalent in special subgroups of patients with CAD.

## Methods

In the current observational study, we prospectively recruited successive patients with angiographically proven significant CAD. The patients presented to the cardiology Department in Alexandria University Hospital because of ischemic heart manifestations in the period between March 2023 and January 2024 and received coronary angiography. CAD was defined as a lumen diameter stenosis of > 70% in ≥ 1 major coronary artery. Previous stroke or transient ischemic attacks were exclusion criteria for the current study. Patients were asked about their willingness to participate in our study. Those who signed an informed consent received carotid ultrasound examination.

Significant CAD was categorized into one of the following groups according to the number of diseased vessels: 1-vessel disease (i.e. patients with disease in 1 vessel), 2-vessel disease (i.e. patients with disease in 2 vessels or left main trunk disease without right coronary artery stenosis), ≥ 3-vessel disease (i.e. patients with disease in 3 vessels or left main trunk disease with right coronary artery stenosis or patients with disease in 3 vessels and the left main trunk disease). The syntax score I was calculated for each patient from the coronary angiographic images (syntaxscore.org). This score quantifies the extent and complexity of CAD by assessing the number of stenotic lesions with ≥ 50% stenosis in vessels ≥ 1.5 mm in diameter. It further encompasses anatomical factors including total occlusions, bifurcations, presence of calcified lesions, vascular tortuosity and lesion length. Atherosclerosis of carotid artery was assessed using duplex ultrasound examination by a single experienced investigator (AE) with ≥ 10 years of experience in neurovascular ultrasound. Plaque assessment was performed blinded to medical history and cardiovascular risk profile. The examination was done for academic research purposes, so that excessive attention was paid for each patient to detect carotid plaques. We examined our patients using the following ultrasound machine: Philips ClearVue 350 (Philips HealthCare, Best, Netherlands) using 4–12 MHz linear array probe. Ultrasound was performed while subjects were lying supine with the head slightly turned away from the side being scanned and the neck slightly extended. We examined the following segments: common carotid artery, carotid bifurcation, internal and external carotid arteries on both sides. The plaque was defined according to the Mannheim Carotid Intima–Media Thickness and Plaque Consensus as a focal lesion in the vessel wall that is at least 1.5 mm thick and protrudes into the vascular lumen [11]. To calculate the plaque score, the carotid artery was divided into 4 segments (common carotid artery, bifurcation, and internal and external carotid artery), and plaques were evaluated in each segment [12]. The diameter of the largest plaque in each segment was measured. Plaque diameters ≥ 1.5, ≥ 2.5, and ≥ 3.5 mm received 1, 2, and 3 points, respectively. The point scores for each of the 4 segments were summarized into a total plaque score, ranging from 0 to 24 points [12]. If a plaque was detected, the degree of stenosis was calculated using the hemodynamic criteria of the North American Symptomatic Carotid Endarterectomy Trial (NASCET) [13]. Hypertension was defined as blood pressure ≥ 140/90 mmHg in at least 2 different measurements or current use of antihypertensive medications [14]. Diabetes mellitus was defined as Hemoglobin A1c ≥ 6.5% or current use of antidiabetic medications [15].

### Statistical analysis

The statistical Package for Social Sciences (SPSS) software version 25 (SPSS, Chicago, IL, USA) was used for data analysis. Continuous variables were expressed as mean ± SD for normally distributed data and as median (interquartile range) for abnormally distributed data. Statistical analysis of categorial data were performed with the χ2 or Fischer exact test (if *n* < 5). Multivariate binary logistic regression analysis was performed to detect independent predictors of carotid artery atherosclerosis with inclusion of variables that showed *p* < 0.1 in the univariate analysis. We conducted a receiver operating curve (ROC) for the relation between syntax score and carotid atherosclerosis. A cut-off value for the syntax score was calculated using the Youden-index. A value of *P* < 0.05 was considered statistically significant.

## Results

**Fig. 1:**
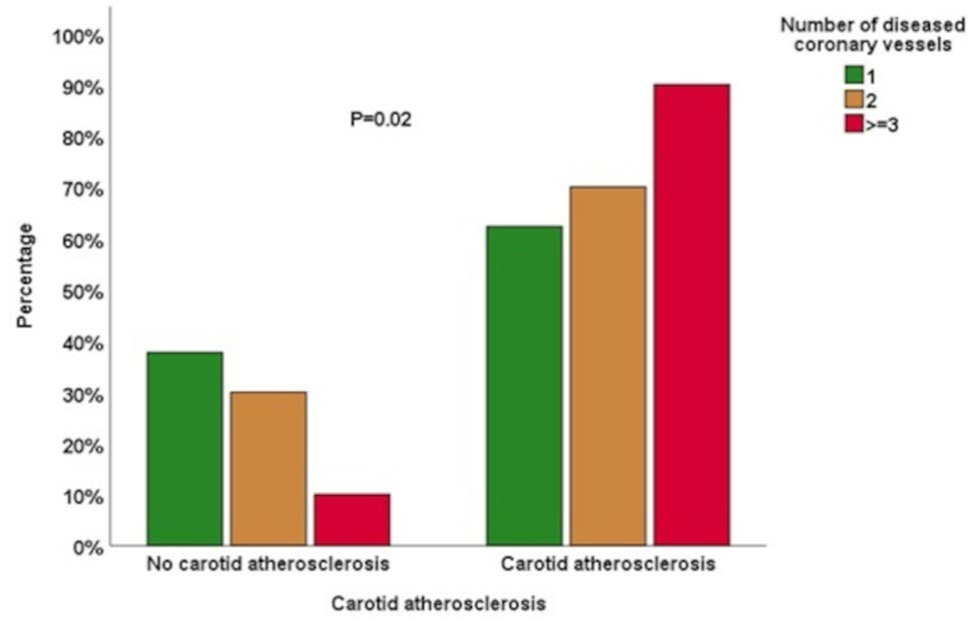
Relation between the number of diseased coronary vessels and carotid atherosclerosis

**Fig. 2:**
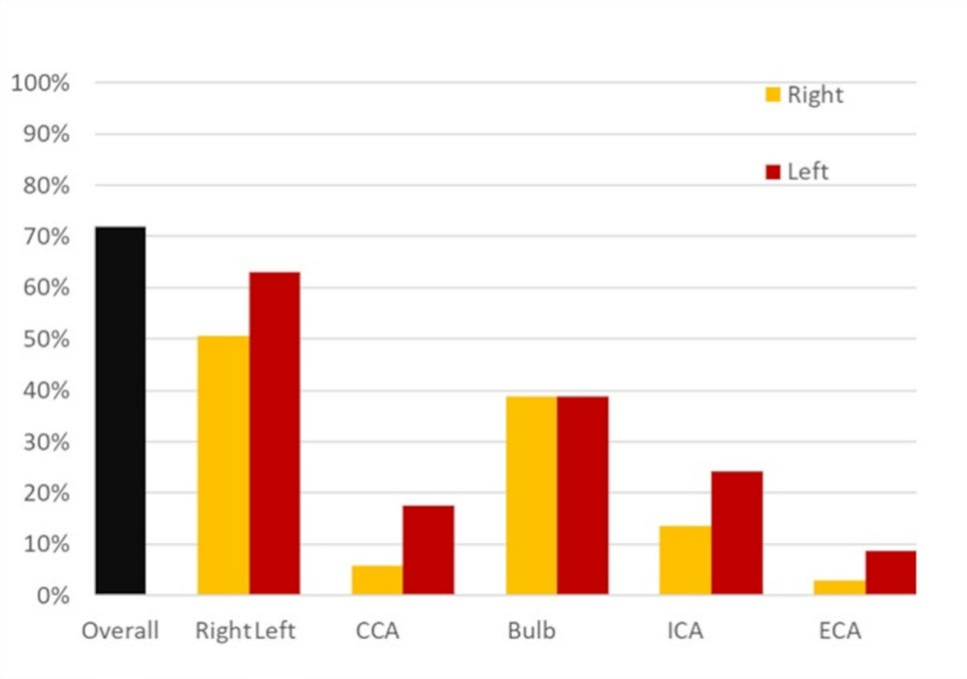
Distribution of atherosclerosis among different carotid segments on both sides (CCA: common carotid artery, ECA: external carotid artery, ICA: internal carotid artery)

**Fig. 3:**
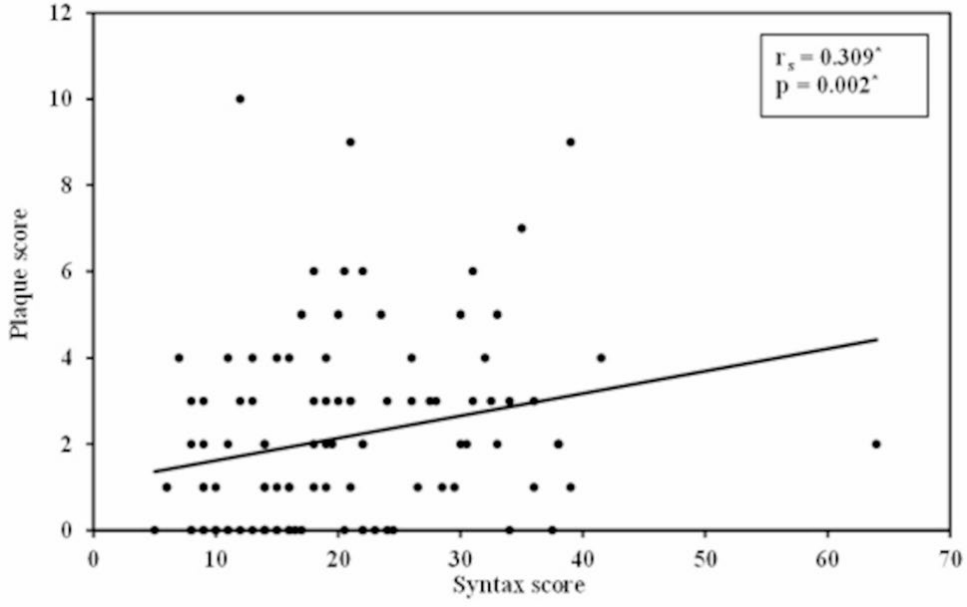
Regression line showing the relation between syntax score and carotid plaque score (*n* = 103)

**Fig. 4:**
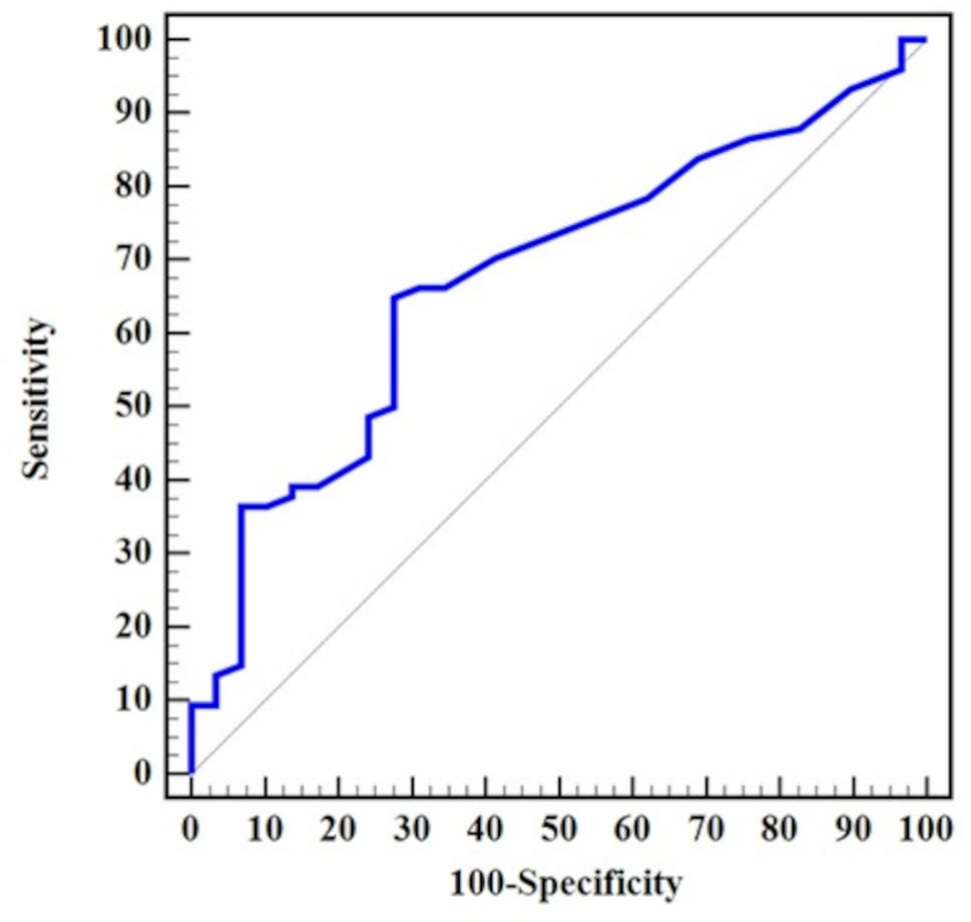
ROC curve showing the relation between Syntax score and carotid atherosclerosis. A cut-off syntax score > 17 yielded a sensitivity of 64.9% and specificity of 72.4% for carotid atherosclerosis (AUC 0.68, 95% CI 0.57–0.79, *p* = 0.006). The cut-off was calculated using the Youden-index

We included 103 patients in our study. Eighty-three (80.6%) of our patients were males, the mean (± SD) age was 58 (± 9) years. The demographic and clinical characteristics of our study population are presented in Table 1 and the comparison of variables between patients with and without carotid atherosclerosis are shown in Table 2. Carotid atherosclerosis was diagnosed in 74 (71.8%) patients. Specifically, 67 (65.1%) patients had non-stenotic plaque, 1 (1%) patient had 20–40% stenosis and 6 (5.8%) patients had ≥ 50% stenosis. Patients with carotid atherosclerosis were older, with a median (IQR) age of 60 (53–67) years, compared to 55 (48–61) years in those without carotid atherosclerosis (*p* = 0.01). The Syntax score was also higher among patients with carotid atherosclerosis, with a median (IQR) of 20 (14–30) versus 14 (11–21) in those without (*p* = 0.006). Non–ST-elevation myocardial infarction (NSTEMI) was more prevalent among patients with carotid atherosclerosis; 20.3% (*n* = 15) of these patients had NSTEMI compared to 3.4% (*n* = 1) of those without carotid atherosclerosis (*p* = 0.04). Carotid atherosclerosis was found in 33 patients (62.3%) with 1-vessel disease, 14 patients (70%) with 2-vessel disease and 27 patients (90%) with ≥ 3-vessel disease (*p* = 0.02) as shown in Fig. 1. The distribution of atherosclerosis across different carotid segments on both sides is illustrated in Fig. 2. Moreover, we observed a statistically significant positive correlation between syntax and plaque score (ρ 0.31, *p* = 0.002). The regression line showing this relation is shown in Fig. 3. In the ROC curve, a syntax score > 17 yielded a sensitivity of 64.9% and specificity of 72.4% for carotid atherosclerosis (AUC 0.68, 95% CI 0.57–0.79, *p* = 0.006) as shown in Fig. 4. Furthermore, we performed a univariate regression model to measure the strength of association between various variables and carotid atherosclerosis as shown in Table 3. Using a multivariate binary logistic regression adjusted for age and sex, we found that age ≥ 60 years (OR 5.1, 95% CI 1.64–15.86, *p* = 0.005), male sex (OR 3.61, 95% CI 1.03–12.67, *p* = 0.045), and presence of ≥ 3-vessel coronary heart disease (OR 4.18, 95% CI 1.06–16.51, *p* = 0.04) were related to carotid atherosclerosis as shown in Table 4. In another age- and sex dependent multivariate logistic regression, syntax score > 17 independently predicted carotid atherosclerosis (OR 4.51, 95% CI 1.68–12.16, *p* = 0.003) as shown in Table 5.

**Table 1:** Baseline characteristics

Charachteristic	No. (%)
Male sex, n (%)	83 (80.6%)
Age (years), median (IQR), mean (± SD)	59 (51–65), 58 (± 9)
Hypertension, n (%)	68 (66%)
Diabetes mellitus, n (%)	55 (53.4%)
Current smoking, n (%)	75 (72.8%)
Chronic coronary syndrome, n (%)	67 (65%)
Unstable angina, n (%)	5 (4.9%)
NSTEMI, n (%)	16 (15.5%)
STEMI, n (%)	54 (52.4%)
History of PAD, n (%)	11 (10.7%)
Syntax score	
median (IQR)	19 (13–27)
Mild (≤ 22), n (%)	69 (67%)
Moderate (23–33), n (%)	21 (20.4%)
Severe (> 33), n (%)	13 (12.6%)
No. of diseased coronary vessels	
1-vessel disease	53 (51.5%)
2-vessel disease	20 (19.4%)
≥3-vessel disease	30 (29.1%)
Carotid plaque score, median (IQR)	2 (0–3)
Carotid atherosclerosis, n (%)	74 (71.8%)
Non-stenotic plaque	67 (65.1)
20–40% stenosis	1 (1%)
50% stenosis	6 (5.8%)

*IQR* Interquartile range, *NSTEMI* Non-ST-Elevation myocardial infarction, *STEMI* ST-Elevation Myocardial infarction, *PAD* Peripheral arterial disease

**Table 2:** Comparison of variables between patients with and without carotid atherosclerosis (n = 103)

Variable	No atherosclerosis (*n* = 29)	Atherosclerosis (*n* = 74)	*P*-valvue
Age in years, median (IQR)	55 (48–61)	60 (53–67)	0.01*
Male sex, n (%)	21 (72.4%)	62 (83.8%)	0.19
Diabetes mellitus, n (%)	15 (51.7%)	40 (54.1%)	0.83
Hypertension, n (%)	17 (58.6%)	51 (68.9%)	0.32
Current smoking, n (%)	19 (65.5%)	56 (75.7%)	0.3
NSTEMI	1 (3.4%)	15 (20.3%)	0.04*
STEMI	19 (65.5%)	35 (47.3%)	0.1
Syntax score	14 (11–21)	20 (14–30)	0.006*

*IQR* Interquartile range, *NSTEMI* Non-ST-Elevation myocardial infarction, *STEMI* ST-Elevation Myocardial infarction

*Statistically significant results

**Table 3:** Univariate logistic regression analysis for the parameters affecting carotid atherosclerosis

	Univariate	
	OR (95%CI)	*p*
Male	1.97 (0.71–5.47)	0.19
Age ≥ 60 years	4.13 (1.57–10.85)	0.004*
Hypertension	1.57 (0.64–3.8)	0.32
Diabetes Mellitus	1.1 (0.47–2.6)	0.83
Current smoking	1.64 (0.65–4.16)	0.30
PAD	4.38 (0.53–35.84)	0.17
Number of diseased coronary vessels		
1-vessel disease	Reference	
2-vessel disease	1.41 (0.47–4.27)	0.54
≥ 3-vessel disease	5.46 (1.46–20.33)	0.01*

CI: Confidence interval, OR: Odd`s ratio, PAD: Peripheral arterial disease

* Statistically significant results

**Table 4:** Multivariate logistic regression analysis for the parameters affecting carotid atherosclerosis

	Multivariate	
	OR (95%CI)	*p*
Male	3.612 (1.03–12.67)	0.045*
Age ≥ 60 years	5.1 (1.64–15.86)	0.005*
Number of diseased coronary vessels		
1-vessel disease	Reference	
2-vessel disease	1.21 (0.37–3.93)	0.75
≥ 3-vessel disease	4.18 (1.06–16.51)	0.04*
Hosmer Lemeshow Test		0.17

*CI* Confidence interval, *OR* Odd`s ratio, *PAD* Peripheral arterial disease

* Statistically significant results

#: Variables with p < 0.1 in the univariate analysis were included in the multivariable regression, with adjustment for age and sex

**Table 5:** Univariate and multivariate logistic regression analysis for the parameters affecting carotid atherosclerosis

	Univariate		Multivariate	
	OR (95%CI)	*P*	OR (95%CI)	*p*
Male sex	1.97 (0.71–5.47)	0.19	3.63 (1-13.27)	0.051
Age ≥ 60 years	4.13 (1.57–10.85)	0.004*	6.2 (1.97–19.51)	0.002*
Syntax score > 17	4.36 (1.73–10.96)	0.002*	4.51 (1.68–12.16)	0.003*
Hosmer-Lemeshow				0.4

*OR* Odd`s ratio, *CI* Confidence interval

* Statistically significant results

## Discussion

Carotid atherosclerosis was highly prevalent in our cohort of patients with coronary artery disease (CAD), with 71.8% showing carotid atherosclerosis and 5.8% having significant (≥ 50%) common or internal carotid artery stenosis. This high burden of carotid disease underscores the close interplay between coronary and carotid atherosclerosis. Moreover, age ≥ 60 years, male sex, a syntax score > 17, and ≥ 3-vessel disease emerged as important predictors of carotid atherosclerosis, highlighting that patients with more advanced or complex CAD may benefit from targeted carotid evaluation and intensified preventive strategies.

A previous literature review reported a mean prevalence of 9% for carotid artery stenosis > 60% among patients with CAD [16], which is slightly higher than the prevalence observed in our cohort. However, the populations included in that review were heterogeneous and represented different ethnic groups, which may partly account for this difference. In line with our results, the authors reported that older age increases the risk for detecting carotid artery stenosis. In a cohort of Egyptian patients with ischemic vascular events, carotid atherosclerosis, common/internal carotid artery stenosis 20–40% and common/internal carotid artery stenosis ≥ 50% were found in 35.8%, 5.2% and 9.9%, respectively [17]. Among our Egyptian patients with CAD, the prevalence of non-stenotic plaques was higher than Egyptian patients with ischemic vascular events in the aforementioned study, yet the prevalence of common/internal carotid artery stenosis ≥ 50% was slightly lower (5.8% in our cohort versus 9.9%). Two other studies from Egypt found internal carotid artery stenosis ≥ 50% among patients with ischemic vascular events in 3.6% [18] and 2.5% [19] of cases, which is lower than the 5.8% observed in our cohort. It’s worth noting that the former study was conducted in Upper Egypt and involved a different ethnic population compared with the Lower Egypt cohort in our study. Additionally, the latter study defined ≥ 50% stenosis based on a peak systolic velocity of ≥ 125 cm/s, whereas our study utilized more recent ultrasound criteria, published after the earlier study, which defines ≥ 50% stenosis as a peak systolic velocity of ≥ 200 cm/s. This updated criterion likely accounts for the higher prevalence observed in our cohort. A study from Korea showed that the prevalence of carotid plaques, defined as > 1.2 mm focal protrusion in the vascular lumen, was 30.3% among patients, who underwent coronary angiography [20]. In the aforementioned study, only 75.4% of the patients with carotid plaques and 58.3% of those without carotid plaques had CAD with luminal stenosis of > 50%. Although the authors used less sensitive criteria for carotid plaques with carotid plaque thickness > 1.2 mm in comparison to the criteria used in our study (≥ 1.5 mm), our cohort showed markedly higher prevalence of carotid plaques (71.8%). However, 100% of our study population had CAD with luminal stenosis ≥ 70%, which might explain the higher prevalence among our cohort. Moreover, ethnic difference might explain the higher prevalence in our study. Similar to our results, a previous Italian cohort detected carotid plaques (defined as increased intimal medial thickness ≥ 1 mm) in 83%, 87%, 89% and 93% of patients with 1-, 2-, 3-vessel CAD and left main trunk CAD, respectively [21]. A more recent Italian Cohort reported similar findings for carotid plaques (defined as focal thickening > 1.5 mm) among 81% of patients with chest pain undergoing coronary angiography (88% had angiographically proven CAD) [22]. Of note, the authors reported a statistically significant relation between syntax score and carotid plaque size, which is comparable with our results. In concordance with our results, other authors reported a positive correlation between carotid plaque size and the number of diseased coronary vessels [23]. In line with our findings, another study from Malaysia among patients with CAD necessitating elective coronary artery bypass surgery reported carotid plaques (defined as focal thickening ≥ 1.3 mm) in 89/119 (74.8%) patients and carotid stenosis (defined as peak systolic velocity ≥ 125 cm/s) in 10/119 (8.4%) patients [24]. Similar to our results, the authors further noted that older age is more likely associated with carotid plaques. In a Norwegian cohort of asymptomatic population aged 63–65 years, the prevalence of carotid plaques was 87% [25], which is even higher than the prevalence (71.8%) in our CAD patients with a mean (± SD) age of 58 (± 9) years. Ethnic differences between Whites and Egyptians might explain the higher prevalence of carotid plaques among Norwegian. Nevertheless, the authors of the Norwegian cohort reported a lower prevalence of > 50% carotid stenosis of 2.3% (defined as peak systolic velocity of > 125 cm/s) in comparison to the prevalence of 5.8% among our patients (defined as peak systolic velocity of ≥ 200 cm/s), which might be explained by the increased cardiovascular risk among our CAD patients.

Overall, ethnic differences, variations in the definition of carotid plaque (> 1 mm, > 1.2 mm, or ≥ 1.5 mm), differences in ultrasound criteria for grading significant carotid stenosis, and varying risk profiles across studies may collectively explain the discrepant findings.

We found a statistically significant positive correlation between syntax score and plaque score (ρ 0.31, *p* = 0.002). Moreover, in our study, a syntax score > 17 was found to be a potential predictor of carotid atherosclerosis, with an area under the curve (AUC) of 0.68, reflecting moderate diagnostic performance. Although this predictive ability is modest, it indicates that patients with more complex coronary artery disease are more likely to have concurrent carotid artery involvement. Clinically, this threshold could help identify patients who may benefit from additional vascular assessment, particularly in settings where carotid imaging is not routinely accessible. However, this cut-off should be applied with caution and requires validation in larger, prospective cohorts. Similar to our findings, a systematic review demonstrated a statistically significant positive correlation between carotid intimal medial thickness and syntax score (*r* = 0.42, *p* < 0.001) as well as between carotid and coronary stenosis (*r* = 0.53, *p* < 0.001) [26]. Another meta-analysis found a positive linear relation between carotid intimal medial thickness and the number of diseased coronary vessels as well as the severity of CAD [27]. In concordance with our study, previous authors reported an association between bilateral rather than unilateral carotid plaques and significant CAD as well as multi-vessel CAD [20].

We found that the presence of ≥ 3-vessel CAD are important predictors of carotid atherosclerosis. Similarly, Tanimoto et al. reported an association between carotid atherosclerosis with age and extent of CAD among Japanese population [28]. Another study from South Korea noted that carotid plaques predicts multi-vessel CAD [20].

Although carotid atherosclerosis is highly prevalent among patients with CAD, atherosclerotic involvement can vary across vascular territories [29]. Routine screening of the carotid arteries may be beneficial for patients who already have CAD. Evidence shows that reducing the carotid intima-media thickness is linked to significantly lower risks of major adverse cardiovascular and major adverse cerebral and coronary events, myocardial infarctions, ischemic strokes and cardiovascular mortality. Conversely, an increase in carotid intima-media thickness is associated with higher risk [30]. Because carotid ultrasound is a simple and widely available tool, targeted follow-up in this high-risk group could help improve risk assessment and guide secondary prevention efforts. However, routine screening for carotid artery disease in people without symptoms is not advised, in line with recommendations from the U.S. Preventive Services Task Force [6]. Therefore, an individualized evaluation is the cornerstone to stratify the cerebro-cardiovascular risk profile and guide patient-tailored management.

The main limitation of the current work is our small sample size (*n* = 103), which limits the generalizability of our findings. However, we recruited our patients after undergoing coronary angiography and conducted ultrasound examination with the aim of detecting possible plaques or stenosis. Because this study used a cross-sectional design, we cannot determine cause-and-effect relationships, so the findings should be viewed as generating hypotheses rather than definitive conclusions. Therefore, any clinical implications—like identifying patients at increased risk for carotid atherosclerosis and complications such as ischemic stroke—should be interpreted carefully. To confirm these results and help develop effective prevention strategies, larger prospective studies with more detailed data are needed. Additionally, the predictive performance of the syntax score > 17 was only moderate (AUC = 0.68), which may limit its clinical utility. Furthermore, the exclusion of asymptomatic CAD patients or those with less severe disease or even physiologically determined lesion severity significantly limits the applicability of the findings to the broader CAD population. Another limitation of our study is that the carotid ultrasound examinations were performed by a single investigator. Although this reduces interobserver variability, it may introduce potential observer bias, and the absence of interobserver validation may affect the reliability of the measurements.

## Conclusions

Ultrasound carotid screening can be considered for patients with CAD, particularly older males with high syntax scores and/or ≥ 3-vessel CAD.

## Data Availability

The datasets used and/or analyzed during the current study are available from the corresponding author on a reasonable request.

## References

[1] Benjamin EJ, Blaha MJ, Chiuve SE, Cushman M, Das SR, Deo R, de Ferranti SD, Floyd J, Fornage M, Gillespie C, et al. Heart disease and stroke Statistics—2017 update: A report from the American heart association. Circulation. 2017;135(10):e146–603. 28122885 10.1161/CIR.0000000000000485 PMC5408160

[2] Pan Y, Jing J, Cai X, Jin Z, Wang S, Wang Y, Zeng C, Meng X, Ji J, Li L, et al. Prevalence and vascular distribution of multiterritorial atherosclerosis among Community-Dwelling adults in Southeast China. JAMA Netw Open. 2022;5(6):e2218307–2218307. 35759265 10.1001/jamanetworkopen.2022.18307 PMC9237794

[3] Alberts MJ, Bhatt DL, Mas JL, Ohman EM, Hirsch AT, Röther J, Salette G, Goto S, Smith SC Jr., Liau CS, et al. Three-year follow-up and event rates in the international reduction of atherothrombosis for continued health registry. Eur Heart J. 2009;30(19):2318–26. 19720633 10.1093/eurheartj/ehp355 PMC2755116

[4] Johnsen SH, Mathiesen EB, Joakimsen O, Stensland E, Wilsgaard T, Løchen M-L, Njølstad I, Arnesen E. Carotid atherosclerosis is a stronger predictor of myocardial infarction in women than in men. Stroke. 2007;38(11):2873–80. 17901390 10.1161/STROKEAHA.107.487264

[5] Herrington W, Lacey B, Sherliker P, Armitage J, Lewington S. Epidemiology of atherosclerosis and the potential to reduce the global burden of atherothrombotic disease. Circul Res. 2016;118(4):535–46. 10.1161/CIRCRESAHA.115.307611 26892956

[6] Jonas DE, Feltner C, Amick HR, Sheridan S, Zheng ZJ, Watford DJ, Carter JL, Rowe CJ, Harris R. U.S. Preventive services task force evidence Syntheses, formerly systematic evidence reviews. Screening for asymptomatic carotid artery stenosis: A systematic review and Meta-Analysis for the US preventive services task force. edn. Rockville (MD): Agency for Healthcare Research and Quality (US); 2014. 25057532

[7] Naylor R, Rantner B, Ancetti S, de Borst GJ, De Carlo M, Halliday A, Kakkos SK, Markus HS, McCabe DJH, Sillesen H’s Choice - European Society for Vascular Surgery (ESVS) 2023 Clinical Practice Guidelines on the Management of Atherosclerotic Carotid and Vertebral Artery Disease, et al. editors. European journal of vascular and endovascular surgery: the official journal of the European Society for Vascular Surgery. 2023;65(1):7-111. 10.1016/j.ejvs.2022.04.011 35598721

[8] Brinjikji W, Rabinstein AA, Lanzino G, Murad MH, Williamson EE, DeMarco JK, Huston J III. Ultrasound characteristics of symptomatic carotid plaques: A systematic review and Meta-Analysis. Cerebrovasc Dis. 2015;40(3–4):165–74. 26279159 10.1159/000437339

[9] O’Leary DH, Polak JF, Kronmal RA, Manolio TA, Burke GL, Wolfson SK Jr. Carotid-artery intima and media thickness as a risk factor for myocardial infarction and stroke in older adults. Cardiovascular health study collaborative research group. N Engl J Med. 1999;340(1):14–22. 9878640 10.1056/NEJM199901073400103

[10] Sillesen H, Muntendam P, Adourian A, Entrekin R, Garcia M, Falk E, Fuster V. Carotid plaque burden as a measure of subclinical atherosclerosis: comparison with other tests for subclinical arterial disease in the high risk plaque bioimage study. JACC: Cardiovasc Imaging. 2012;5(7):681–9. 22789936 10.1016/j.jcmg.2012.03.013

[11] Touboul PJ, Hennerici MG, Meairs S, Adams H, Amarenco P, Desvarieux M, Ebrahim S, Fatar M, Hernandez Hernandez R, Kownator S, et al. Mannheim intima-media thickness consensus. Cerebrovasc Dis. 2004;18(4):346–9. 15523176 10.1159/000081812

[12] ten Kate GL, ten Kate GJ, van den Oord SC, Dedic A, Dharampal AS, Nieman K, de Feyter PJ, Sijbrands EJ, van der Steen AF, Schinkel AF. Carotid plaque burden as a measure of subclinical coronary artery disease in patients with heterozygous Familial hypercholesterolemia. Am J Cardiol. 2013;111(9):1305–10. 23411100 10.1016/j.amjcard.2013.01.274

[13] Arning C, Widder B, von Reutern GM, Stiegler H, Gortler M. Revision of DEGUM ultrasound criteria for grading internal carotid artery stenoses and transfer to NASCET measurement. Ultraschall Der Medizin (Stuttgart Germany: 1980). 2010;31(3):251–7. 10.1055/s-0029-1245336 20414854

[14] Meschia JF, Bushnell C, Boden-Albala B, Braun LT, Bravata DM, Chaturvedi S, Creager MA, Eckel RH, Elkind MS, Fornage M, et al. Guidelines for the primary prevention of stroke: a statement for healthcare professionals from the American Heart Association/American Stroke Association. Stroke. 2014;45(12):3754–832. 25355838 10.1161/STR.0000000000000046 PMC5020564

[15] Klink T, Geiger J, Both M, Ness T, Heinzelmann S, Reinhard M, Holl-Ulrich K, Duwendag D, Vaith P, Bley TA. Giant cell arteritis: diagnostic accuracy of MR imaging of superficial cranial arteries in initial diagnosis-results from a multicenter trial. Radiology. 2014;273(3):844–52. 25102371 10.1148/radiol.14140056

[16] Aboyans V, Lacroix P. Indications for carotid screening in patients with coronary artery disease. La Presse Médicale. 2009;38(6):977–86. 19376684 10.1016/j.lpm.2009.02.015

[17] Elhfnawy A, Galeel AA, Abdelkhalek H. Carotid atherosclerosis in a sample of Egyptian patients with or without ischemic vascular events. Egypt J Neurol Psychiatry Neurosurg. 2023;59(1):144.

[18] Soliman RH, Oraby MI, Fathy M, Essam AM. Risk factors of acute ischemic stroke in patients presented to Beni-Suef university hospital: prevalence and relation to stroke severity at presentation. Egypt J Neurol Psychiatr Neurosurg. 2018;54(1):8. 29780228 10.1186/s41983-018-0012-4 PMC5954772

[19] Abd Allah F, Baligh E, Ibrahim M. Clinical relevance of carotid atherosclerosis among egyptians: a 5-year retrospective analysis of 4,733 subjects. Neuroepidemiology. 2010;35(4):275–9. 20881431 10.1159/000319899

[20] Kwon TG, Kim KW, Park HW, Jeong JH, Kim KY, Bae JH. Prevalence and significance of carotid plaques in patients with coronary atherosclerosis. Korean Circulation J. 2009;39(8):317–21. 19949637 10.4070/kcj.2009.39.8.317 PMC2771847

[21] Ambrosetti M, Casorati P, Salerno M, Zambelli M, Pedretti RF, Tramarin R. Newly diagnosed carotid atherosclerosis in patients with coronary artery disease admitted for cardiac rehabilitation. Italian Heart Journal: Official J Italian Federation Cardiol. 2004;5(11):840–3. 15633439

[22] Migliorino D, Mignano A, Evola S, Polizzi G, Novo G, Corrado E, Novo S. Correlation between carotid atherosclerosis and coronary artery disease: A retrospective study of 1067 patients. Nutr Metabolism Cardiovasc Dis. 2017;27(1):e28.

[23] Sugioka K, Hozumi T, Iwata S, Oe H, Okuyama T, Shirai N, Yamashita H, Ehara S, Kataoka T, Yoshikawa J, et al. Morphological but not functional changes of the carotid artery are associated with the extent of coronary artery disease in patients with preserved left ventricular function. Stroke. 2008;39(5):1597–9. 18340102 10.1161/STROKEAHA.107.502732

[24] Sahadevan M, Chee KH, Tai MS. Prevalence of extracranial carotid atherosclerosis in the patients with coronary artery disease in a tertiary hospital in Malaysia. Medicine. 2019;98(15):e15082. 30985661 10.1097/MD.0000000000015082 PMC6485885

[25] Ihle-Hansen H, Vigen T, Ihle-Hansen H, Rønning OM, Berge T, Thommessen B, Lyngbakken MN, Orstad EB, Enger S, Nygård S et al. Prevalence of carotid plaque in a 63- to 65-Year-Old Norwegian cohort from the general population: the ACE (Akershus cardiac Examination) 1950 study. J Am Heart Association. 2018;7(10):e008562. 10.1161/JAHA.118.008562. 10.1161/JAHA.118.008562 PMC6015330 29739796

[26] Bytyçi I, Shenouda R, Wester P, Henein MY. Carotid atherosclerosis in predicting coronary artery disease. Arterioscler Thromb Vasc Biol. 2021;41(4):e224–37. 33626907 10.1161/ATVBAHA.120.315747

[27] Shenouda R, Bytyci I, Henein MY. Carotid and coronary atherosclerosis: A systematic review and meta-analysis. Atherosclerosis. 2021;331:e105. 10.1161/ATVBAHA.120.315747 33626907

[28] Tanimoto S, Ikari Y, Tanabe K, Yachi S, Nakajima H, Nakayama T, Hatori M, Nakazawa G, Onuma Y, Higashikuni Y, et al. Prevalence of carotid artery stenosis in patients with coronary artery disease in Japanese population. Stroke. 2005;36(10):2094–8. 16179563 10.1161/01.STR.0000185337.82019.9e

[29] Piechocki M, Przewłocki T, Pieniążek P, Trystuła M, Podolec J, Kabłak-Ziembicka A. A Non-Coronary, peripheral arterial atherosclerotic disease (Carotid, Renal, lower Limb) in elderly patients—A review PART II—Pharmacological approach for management of elderly patients with peripheral atherosclerotic lesions outside coronary territory. J Clin Med. 2024;13(5):1508. 38592348 10.3390/jcm13051508 PMC10934701

[30] Gacoń J, Przewłocki T, Podolec J, Badacz R, Pieniążek P, Mleczko S, Ryniewicz W, Żmudka K, Kabłak-Ziembicka A. Prospective study on the prognostic value of repeated carotid intima-media thickness assessment in patients with coronary and extra coronary steno-occlusive arterial disease. Pol Archives Intern Med. 2019;129(1):12–21. 10.20452/pamw.4407 30600311

